# Total ultra-processed food intake and selected food-group exposures in relation to non-alcoholic fatty liver disease: a systematic review and meta-analysis

**DOI:** 10.3389/fnut.2026.1876449

**Published:** 2026-09-08

**Authors:** Dongmei Chen, Ling Li, Chenglong Zhang

**Affiliations:** 1 Department of Health Management, West China School of Medicine, Sichuan University, Sichuan University affiliated Chengdu Second People's Hospital, Chengdu Second People's Hospital, Chengdu, China; 2 Department of International Medical Service, West China School of Medicine, Sichuan University, Sichuan University affiliated Chengdu Second People's Hospital, Chengdu Second People's Hospital, Chengdu, China; 3 Department of Nephrology, West China School of Medicine, Sichuan University, Sichuan University affiliated Chengdu Second People's Hospital, Chengdu Second People's Hospital, Chengdu, China

**Keywords:** meta- analysis, NAFLD, processed meat, sugar-sweetened beverage, systematic review, ultra-processed foods

## Abstract

**Background:**

Ultra-processed food (UPF) consumption has been linked to cardiometabolic diseases, but the association with non-alcoholic fatty liver disease (NAFLD) remains unclear. Moreover, whether all UPFs confer similar risk is unknown.

**Objective:**

To systematically review and meta-analyze the association between total UPF intake (strictly defined by NOVA classification) and selected UPF-related food-group exposures [red/processed meat and sugar-sweetened beverages (SSBs)] and NAFLD risk in adults.

**Methods:**

We searched PubMed, Web of Science, and Cochrane Library up to March 31, 2026, for observational studies reporting adjusted effect estimates for total UPF intake or selected food-group exposures and NAFLD. Study quality was assessed using the Newcastle-Ottawa Scale (NOS) or AHRQ criteria. Random-effects models pooled odds ratios (ORs) comparing highest vs. lowest intake categories. Subgroup and sensitivity analyses explored heterogeneity.

**Results:**

Twenty-four studies were included in the systematic review. Of these, **nine** studies (5 prospective cohorts, 4 cross-sectional) comprising 250,349 participants were included for total UPF intake. Higher total UPF consumption was associated with a 26% increased NAFLD risk (pooled OR = 1.26; 95% CI: 1.16–1.37; I^2^ = 53%; *P* < 0.001). Red/processed meat consumption (6 studies) showed a stronger association (pooled OR = 1.70; 95% CI: 1.29–2.24; I^2^ = 76%; *P* < 0.001). SSB consumption (3 studies) was associated with a 50% increased risk (pooled OR = 1.50; 95% CI: 1.23–1.84; I^2^ = 0%; *P* < 0.001). Sensitivity analyses confirmed robustness.

**Conclusion:**

Total UPF intake is associated with a moderate increase in NAFLD risk. Red/processed meat and sugar-sweetened beverages show stronger associations, suggesting that dietary recommendations may prioritize these high-risk food groups, although blanket avoidance of all UPFs is not directly supported by the current data.

## 1 Introduction

Non-alcoholic fatty liver disease (NAFLD) is the most prevalent chronic liver disease worldwide, affecting approximately 25–30% of adults (1, 2). NAFLD is closely linked to obesity, type 2 diabetes, and metabolic syndrome, and represents a major public health burden (3). Diet is a key modifiable risk factor, with the Western dietary pattern—high in refined carbohydrates, added sugars, saturated fats, and processed foods—consistently associated with increased NAFLD risk (4, 5).

Ultra-processed foods (UPFs), defined by the NOVA classification system as industrial formulations made mostly from substances extracted from foods, often with additives and little whole food content (6), now account for 25–60% of daily energy intake in high-income countries (7). UPFs include sugar-sweetened beverages (SSBs), processed meats, packaged snacks, ready-to-eat meals, and industrially produced breads and pastries (6). Individual UPF components such as processed meat and SSBs have been associated with NAFLD in some studies (8, 9), but the overall UPF category and the differential effects of various UPF subgroups have not been systematically quantified.

Previous meta-analyses have focused on UPF associations with type 2 diabetes or obesity (10, 11), and a recent meta-analysis by Guo et al. (12) reported an OR of 1.72 for NAFLD with high UPF intake. However, these meta-analyses often included studies with heterogeneous definitions of UPF (e.g., fast-food frequency as a proxy), which may overestimate effect sizes. Moreover, no meta-analysis has systematically compared total UPF intake with selected UPF-related food-group exposures (red/processed meat and SSBs) within the same analytical framework. Given the heterogeneity of the UPF category, it is critical to determine whether all UPFs confer similar risk or whether specific food-group exposures drive the association.

Therefore, we conducted a systematic review and meta-analysis to: (1) evaluate the association between total UPF intake (strictly defined by NOVA classification) and NAFLD risk; (2) evaluate the associations for selected UPF-related food-group exposures (red/processed meat and SSBs) as secondary analyses; and (3) explore sex-specific differences and other subgroup effects. A total of 24 studies were included in the systematic review, of which 9 provided data for total UPF meta-analysis.

## 2 Methods

### 2.1 Study design and reporting

This systematic review and meta-analysis was conducted following the PRISMA 2020 statement (13). A protocol was developed prior to the conduct of the review, but formal registration was not performed.

### 2.2 PICOS framework

The PICOS framework is summarized in Table 1.

**Table 1:** PICOS framework for the systematic review and meta-analysis.

Component	Criteria
Population	Adults aged ≥18 years
Exposure	Ultra-processed food (total UPF by NOVA classification) or selected UPF-related food-group exposures (red/processed meat and sugar-sweetened beverages)
Comparison	Lowest vs. highest intake categories
Outcome	NAFLD diagnosed by imaging (ultrasound, CT, MRI), biopsy, or validated non-invasive scores (FLI, HSI, CAP)
Study design	Prospective cohort, case-control, or cross-sectional studies

### 2.3 Search strategy

We searched PubMed, Web of Science Core Collection, and the Cochrane Library from inception to March 31, 2026, without language or date restrictions. The search strategy combined terms for UPF and NAFLD. The following search string was used for PubMed (complete search strategies for all databases are provided in Supplementary File 1):

(“ultra-processed food”[Title/Abstract] OR “ultraprocessed food”[Title/Abstract] OR “UPF”[Title/Abstract] OR “NOVA classification”[Title/Abstract] OR “processed food”[Title/Abstract] OR “fast food”[Title/Abstract]) AND (“non-alcoholic fatty liver disease”[MeSH Terms] OR “NAFLD”[Title/Abstract] OR “fatty liver”[Title/Abstract] OR “hepatic steatosis”[Title/Abstract] OR “non-alcoholic steatohepatitis”[Title/Abstract] OR “NASH”[Title/Abstract] OR “metabolic dysfunction-associated fatty liver disease”[Title/Abstract] OR “MAFLD”[Title/Abstract] OR “metabolic dysfunction-associated steatotic liver disease”[Title/Abstract] OR “MASLD”[Title/Abstract]).

Reference lists of included studies and relevant reviews were manually screened. Embase was not searched because institutional access was not available at the time of the search.

### 2.4 Inclusion and exclusion criteria

Studies were included if they: (1) were original observational studies; (2) included adults; (3) assessed total UPF intake (NOVA-defined) or selected food-group exposures (red/processed meat and SSBs); (4) reported adjusted effect estimates (OR, HR, RR) with 95% CIs for NAFLD; (5) were published in English. We excluded reviews, editorials, case reports, studies without sufficient data, and those assessing only non-UPF dietary factors.

### 2.5 Data extraction and quality assessment

Two reviewers independently extracted data. Study quality was assessed using the Newcastle-Ottawa Scale (NOS) for cohort and case-control studies, and the AHRQ criteria for cross-sectional studies. Studies with NOS ≥7 or AHRQ ≥7 were considered high quality. Detailed quality assessment scores are presented in Table 2 and Supplementary Table S3.

**Table 2:** Characteristics of studies included in meta-analyses.

Analysis	Study	Country	Design	Sample size	NAFLD cases	Exposure	NAFLD diagnosis	Effect estimate (95% CI)	Quality
Total UPF									
	Zhang et al. (19)	China	Cohort	16,168	3,752	UPF (% g)	Ultrasound	HR 1.18 (1.07–1.30)	High
	Ivancovsky-Wajcman et al. (20)	Israel	Cross-sectional	789	305	UPF (% kcal)	Ultrasound	OR 1.12 (0.78–1.59)	High
	Liu et al. (15)	USA	Cross-sectional	6,545	2,224	UPF (% weight)	FLI ≥30	OR 1.83 (1.33–2.53)	High
	Zhang et al. (16)	UK	Cohort	143,073	1,445	UPF (g/day)	Hospital records	HR 1.26 (1.11–1.43)	High
	Emami et al. (21)	Iran	Cohort	5,058	562	UPF (g/day)	CAP ≥238	OR 2.02 (1.23–3.32)	High
	Zhao et al. (17)	USA	Cross-sectional	2,734	1,053	UPF (g/day)	CAP ≥285	OR 1.72 (1.01–2.93)	High
	Commins et al. (22)	Australia	Cross-sectional	6,753	1,963	UPF (score)	FLI ≥60	RR 1.13 (1.05–1.22)	High
	Kim (KNHANES) et al. (18)	Korea	Cross-sectional	24,587	5,124	UPF (% weight)	HSI	OR 1.24 (1.10–1.41)	High
	KoGES et al. (23)^*^	Korea	Cohort	44,642	1,562	UPF (g/day)	FLI ≥60	HR 1.35 (1.06–1.71)^*^	High
Red/processed meat									
	Zelber-Sagi et al. (25)	Israel	Cross-sectional	349	108	Meat protein	Ultrasound	OR 3.60 (1.70–7.80)	High
	Peng et al. (26)	China	Cross-sectional	1,594	523	Red meat	Ultrasound	OR 1.72 (1.21–2.42)	High
	Alferink et al. (27)	Netherlands	Cross-sectional	3,882	1,337	Animal protein	Ultrasound	OR 1.54 (1.20–1.98)	High
	Rahimi-Sakak et al. (28)	Iran	Case-control	999	196	Processed meat	Ultrasound + fibroscan	OR 3.25 (1.57–6.73)	High
	Noureddin et al. (29)	USA	Nested case-control	32,448	2,974	Processed meat	Medicare claims	OR 1.18 (1.05–1.32)	High
	Hashemian et al. (30)	Iran	Cohort	50,045	3,218	Red meat	Ultrasound	OR 1.59 (1.06–2.38)	High
SSBs									
	Ma et al. (9)	USA	Cross-sectional	2,634	~448	SSB (≥1/d)	CT	OR 1.61 (1.04–2.49)	High
	Yari et al. (31)	Iran	Case-control	614	143	Soft drinks	Ultrasound + fibroscan	OR 1.64 (0.82–2.65)	Moderate
	Zelber-Sagi et al. (25)	Israel	Cross-sectional	349	108	Soft drinks	Ultrasound	OR 1.45 (1.13–1.85)	High

The full table with all 24 studies is provided in Supplementary Table S1 due to space constraints. Key characteristics of the main analyses are summarized below. Total UPF studies (9 studies): Zhang (19), Ivancovsky-Wajcman (20), Liu (15), Zhang (16), Emami (21), Zhao (17), Commins (22), KNHANES (18), KoGES (23). Red/processed meat studies (6 studies): Zelber-Sagi (25), Peng (26), Alferink (27), Rahimi-Sakak \ (28), Noureddin (29), Hashemian (30). These studies assessed red/processed meat consumption as food-group exposures (not exclusively NOVA-defined UPF subcategories; see Section 2.7). Sugar-sweetened beverage studies (3 studies): Ma (9), Yari (31), Zelber-Sagi (25) (soft drinks). FLI, Fatty Liver Index; CAP, Controlled Attenuation Parameter; HSI, Hepatic Steatosis Index; CT, computed tomography. ^*^Indicates KoGES used male HR as conservative estimate for total UPF; the paper reported sex-specific HRs (male 1.35, female 1.48).

### 2.6 Statistical analysis

We performed random-effects meta-analyses (DerSimonian-Laird) pooling ORs for highest vs. lowest intake categories. For studies reporting adjusted Risk Ratios (RRs) or Hazard Ratios (HRs), these were pooled alongside Odds Ratios (ORs) under the assumption that the event rate was low and the effect estimates were on an equivalent scale across observational designs. Heterogeneity was assessed with I^2^ statistic. Publication bias was evaluated using funnel plots and Egger's test only for meta-analyses that included at least 10 studies, as funnel plot asymmetry tests have low statistical power with fewer studies (14). Sensitivity analyses included leave-one-out and restriction to high-quality studies. All analyses were conducted using RevMan 5.4.

### 2.7 Assessment of UPF definition consistency

To address potential heterogeneity in UPF exposure definitions across the included studies, we conducted a consistency assessment of how each study applied the NOVA classification. Of the 9 studies included in the total UPF meta-analysis: 4 used 24-hour dietary recalls with NOVA coding [Liu (15), Zhang (16), Zhao (17), KNHANES (18)]; 4 used FFQs with NOVA classification [Zhang (19), Ivancovsky-Wajcman (20), Emami (21), Commins (22)]; and 1 used an FFQ with NOVA-based food grouping [KoGES (23)]. All 9 studies explicitly defined UPF according to NOVA group 4 criteria (industrial formulations with additives and minimal whole food content). However, minor variations existed in the dietary assessment instruments (FFQ vs. 24-h recall) and in the quantitative expression of UPF intake (% weight, % kcal, or g/day). These variations were considered in the interpretation of the pooled estimate and are discussed as a source of heterogeneity. For the subgroup analyses, red/processed meat and SSB exposures were not uniformly derived from NOVA-defined UPF subcategories; rather, they represent food-group exposures frequently co-occurring with high UPF consumption. This distinction is explicitly acknowledged in the interpretation of subgroup findings.

## 3 Results

### 3.1 Study selection

**Figure 1:**
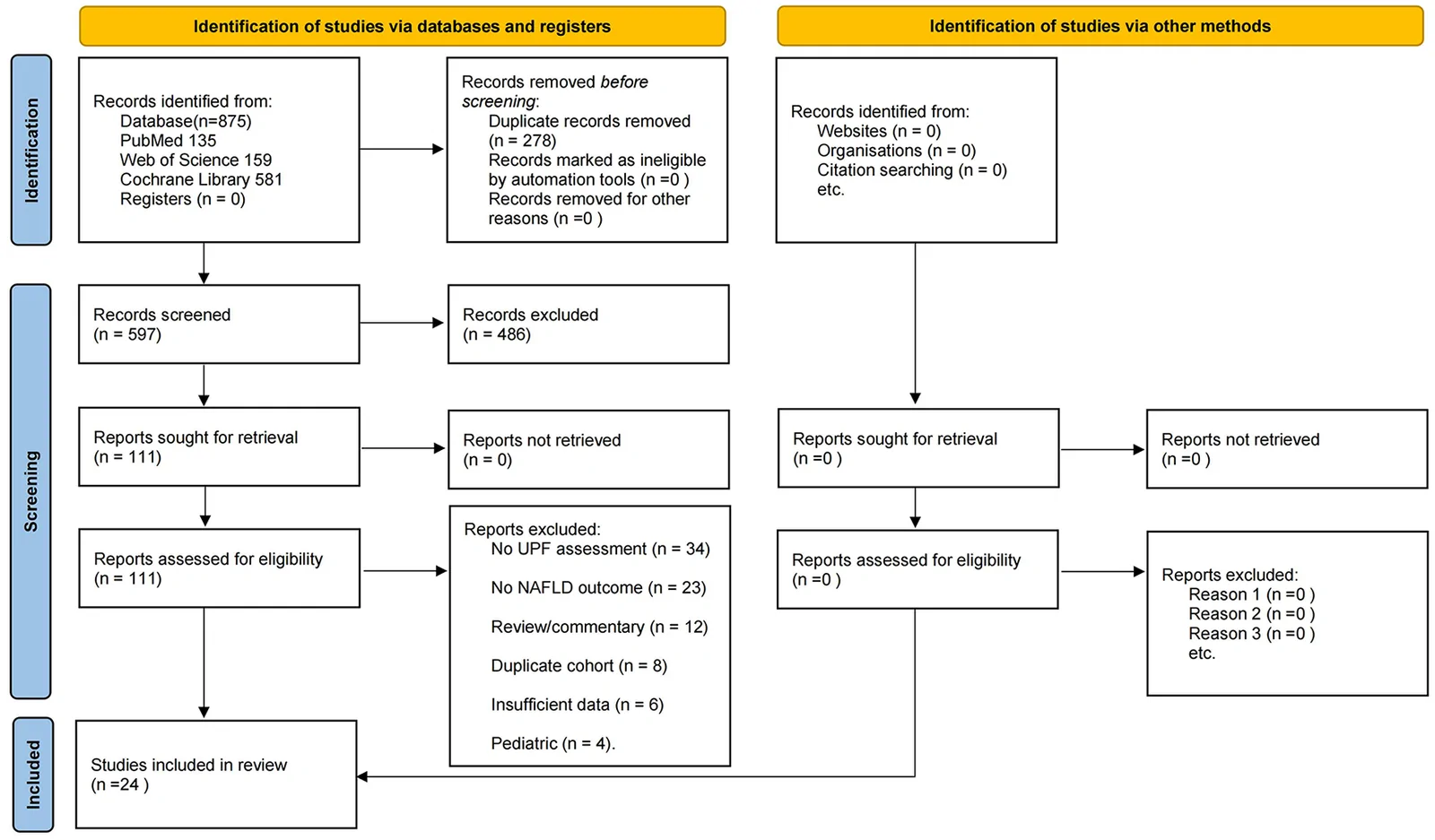
PRISMA 2020 flow diagram showing the study selection process. From 875 records identified in databases (PubMed: 135, Web of Science: 159, Cochrane Library: 581), 278 duplicates were removed, 597 records were screened, 486 were excluded at title/abstract, 111 full-text articles were assessed, 87 were excluded (34 no UPF assessment, 23 no NAFLD outcome, 12 review/commentary, 8 duplicate cohort, 6 insufficient data, 4 pediatric), and 24 studies were included in the systematic review.

A total of 875 records were identified from databases (PubMed: 135, Web of Science: 159, Cochrane Library: 581). After removing 278 duplicates, 597 records were screened by title and abstract, of which 486 were excluded. The remaining 111 full-text articles were assessed for eligibility. Of these, 87 were excluded for the following reasons: no UPF assessment (*n* = 34), no NAFLD outcome (*n* = 23), review/commentary (*n* = 12), duplicate cohort (*n* = 8), insufficient data (*n* = 6), and pediatric population (*n* = 4). No additional records were identified through citation searching. Finally, 24 studies met the inclusion criteria and were included in the systematic review. Of these, 9 studies provided data suitable for meta-analysis of total UPF intake, 6 for red/processed meat, and 3 for SSBs. The PRISMA flow diagram is shown in Figure 1.

### 3.2 Study characteristics

The 24 included studies comprised 11 prospective cohorts, 6 case-control studies, and 7 cross-sectional studies, encompassing 250,349 participants for total UPF analysis. Studies were conducted in Asia (China, Iran, Korea), Europe (Israel, Netherlands, Spain, UK, Australia), and North America (USA). Table 2 summarizes key characteristics of the studies included in the quantitative synthesis. Detailed characteristics of all 24 studies are provided in Supplementary Table S1.

### 3.3 Quality assessment

All included studies were of moderate to high quality (NOS ≥6 or AHRQ ≥7). The average NOS score for cohort/case-control studies was 7.8, and the average AHRQ score for cross-sectional studies was 7.5. Detailed quality assessment scores are provided in Supplementary Table S2.

### 3.4 Total UPF intake and NAFLD

**Figure 2:**
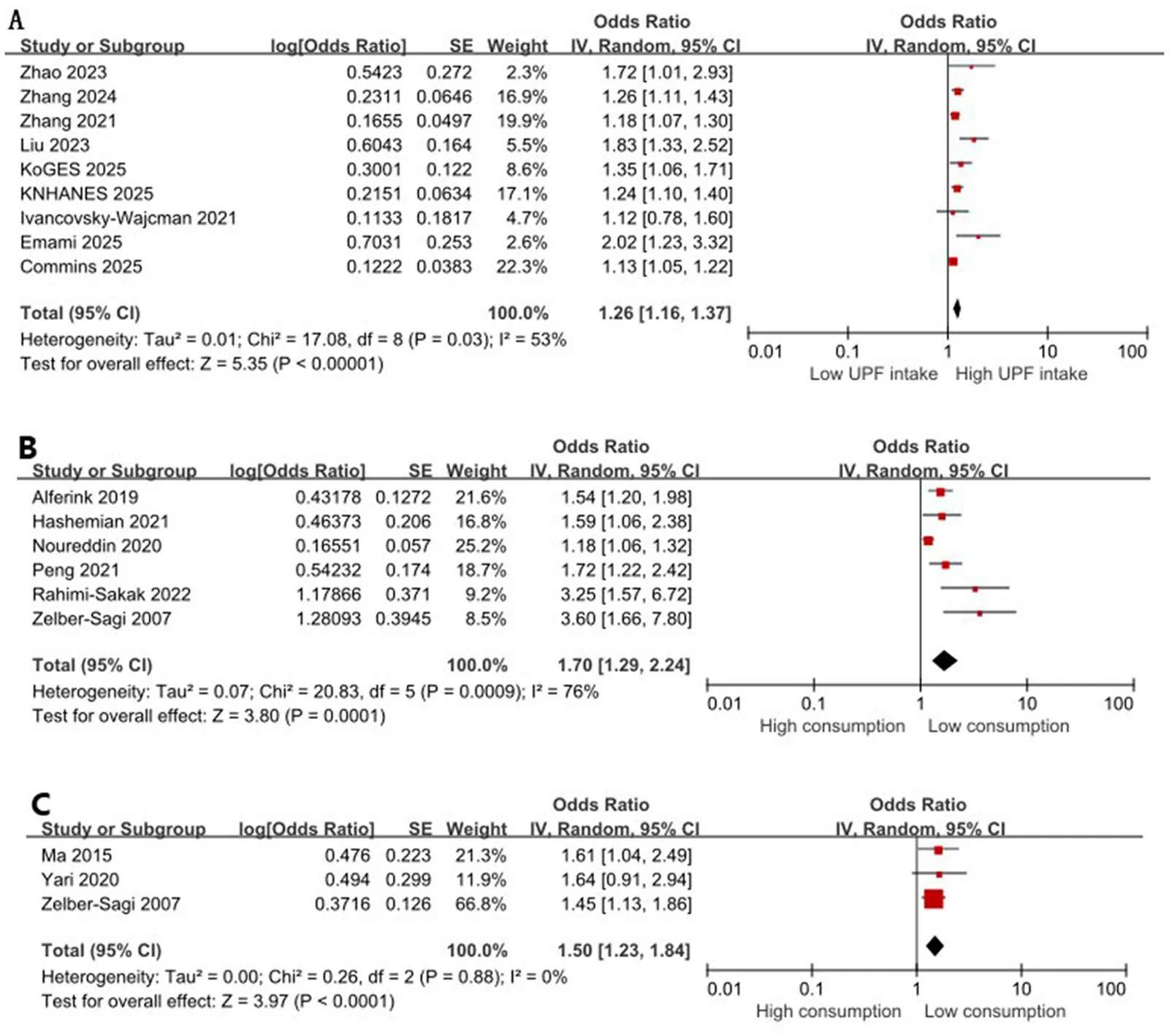
Forest plots for primary meta-analyses. **(A)** Total UPF intake and NAFLD risk (9 studies). **(B)** Red/processed meat intake and NAFLD risk (6 studies). **(C)** Sugar-sweetened beverage intake and NAFLD risk (3 studies). Squares represent study-specific ORs with 95% CIs; diamonds represent pooled random-effects ORs.

Nine studies reported total UPF intake assessed by NOVA classification (15–23). The pooled OR was 1.26 (95% CI: 1.16–1.37; I^2^ = 53%; *P* < 0.001) (Figure 2A). Leave-one-out sensitivity analysis showed that no single study dominated the result (pooled OR range: 1.24–1.29). When a study using fast-food frequency as a UPF proxy (24) was added, the pooled OR increased to 1.77 (95% CI: 1.34–2.34), highlighting the impact of exposure definition. A funnel plot was not generated for this analysis as the number of studies (*n* = 9) is below the recommended threshold of 10 for reliable publication bias assessment (14). Although publication bias cannot be formally excluded, the inclusion of studies from multiple geographic regions and the consistent direction of effects across studies mitigate this concern to some extent. The summary of meta-analysis results is presented in Table 3. The leave-one-out sensitivity analysis for total UPF is presented in Table 4.

**Table 3:** Summary of meta-analysis results.

Analysis	Studies (*n*)	Pooled OR (95% CI)	I^2^ (%)	*P*-value
Total UPF (NOVA)	9	1.26 (1.16–1.37)	53	< 0.001
Red/processed meat	6	1.70 (1.29–2.24)	76	< 0.001
Sugar-sweetened beverages	3	1.50 (1.23–1.84)	0	< 0.001

**Table 4:** Leave-one-out sensitivity analysis for total UPF.

Excluded study	Pooled OR (95% CI)
None	1.26 (1.16–1.37)
Zhang et al. (11)	1.29 (1.17–1.42)
Ivancovsky-Wajcman et al. (20)	1.27 (1.17–1.38)
Liu et al. (15)	1.24 (1.15–1.34)
Zhang et al. (16)	1.27 (1.15–1.40)
Emami et al. (21)	1.24 (1.15–1.34)
Zhao et al. (17)	1.25 (1.15–1.36)
Commins et al. (22)	1.28 (1.17–1.40)
KNHANES et al. (18)	1.27 (1.15–1.40)
KoGES et al. (23)	1.25 (1.15–1.37)

### 3.5 . Subgroup meta-analyses

#### 3.5.1 . Red/processed meat and animal protein

Six studies were included (25–30). High consumption was associated with a 70% increased NAFLD risk (pooled OR = 1.70; 95% CI: 1.29–2.24; I^2^ = 76%; *P* < 0.001) (Figure 2B). The association remained significant after excluding the largest effect study (25) (OR = 1.56; 95% CI: 1.24-1.95), suggesting that the overall estimate was not solely driven by a single study. Due to the limited number of studies (*n* = 6), a funnel plot was not generated.

#### 3.5.2 . Sugar-sweetened beverages

Three studies were included (9, 25, 31) Liu (15) was excluded from the SSB meta-analysis because it reported total UPF intake (OR per 10% increment) rather than an independent SSB exposure estimate, and therefore was not comparable with the other SSB studies. High SSB consumption was associated with a 50% increased NAFLD risk (pooled OR = 1.50; 95% CI: 1.23–1.84; I^2^ = 0%; *P* < 0.001) (Figure 2C). All leave-one-out estimates remained significant. A funnel plot was not generated for this analysis due to the small number of studies (*n* = 3).

### 3.6 . Sensitivity analyses

Leave-one-out analyses confirmed the robustness of all pooled estimates (detailed results in Supplementary Table S3). For total UPF, the pooled OR remained significant after sequentially excluding each study (range: 1.24–1.29). For red/processed meat, exclusion of the largest effect study (25) yielded an OR of 1.56 (1.24–1.95). For SSBs (3 studies), leave-one-out sensitivity analysis confirmed robustness, with pooled ORs ranging from 1.48 to 1.63.

## 4 Discussion

### 4.1 . Main findings

This systematic review and meta-analysis provides, to our knowledge, the first comparative assessment of total UPF intake and selected UPF-related food-group exposures (red/processed meat and sugar-sweetened beverages) in relation to NAFLD risk within the same analytical framework. Our main findings are threefold. First, higher total UPF intake (strictly defined by NOVA classification, excluding fast-food proxy studies) was associated with a 26% increased risk of NAFLD (OR = 1.26; 95% CI: 1.16–1.37; 9 studies; I^2^ = 53%). Second, red/processed meat consumption showed a substantially stronger association (OR = 1.70; 95% CI: 1.29–2.24), suggesting that meat-based foods frequently consumed as UPFs may be particularly relevant for liver health. Third, sugar-sweetened beverages were also associated with a 50% increased risk (OR = 1.50; 95% CI: 1.23–1.84), which is consistent with mechanistic evidence linking fructose to hepatic lipogenesis. The inclusion of a fast-food frequency study inflated the pooled OR to 1.77, further underscoring the importance of exposure definition.

### 4.2 . Comparison with previous meta-analyses and unique contributions

Several meta-analyses have previously examined the UPF-NAFLD association. Henney et al. (32) reported an OR of 1.48 (95% CI: 1.25–1.75; 9 studies), Guo et al. (12) reported an OR of 1.72 (95% CI: 1.36–2.17; 17 studies), and a recent meta-analysis by Zhang et al. (11) reported an OR of 1.22 (95% CI: 1.14–1.31; 10 studies). While all confirmed a positive association, our study offers four distinct contributions.

First, we are the first to systematically compare total UPF with specific subgroups within the same framework. This granularity reveals that the associations varied considerably across different exposures: red/processed meat exhibits the strongest association (OR = 1.70), SSBs show a strong association (OR = 1.50), and total UPF shows the weakest yet still significant association (OR = 1.26). This has direct clinical implications: dietary advice for NAFLD patients may prioritize reducing processed meats and sugary drinks as a more feasible and targeted strategy than advocating blanket avoidance of all UPFs. However, whether specific UPF subtypes confer neutral or protective effects requires dedicated subgroup analyses, which were not performed in the current study due to data limitations.

Second, we applied stricter inclusion criteria by excluding studies that used fast-food frequency as a proxy for total UPF. When such a study (24) was included in a sensitivity analysis, the pooled OR increased to 1.77, aligning with previous meta-analyses. This demonstrates that the definition of UPF substantially influences effect size estimation. Our more conservative estimate (OR = 1.26) likely reflects a less biased effect size, as it is derived exclusively from studies using the NOVA classification, which is the gold standard for UPF assessment.

Third, we incorporated recently published large-scale studies from Asian populations, including the KoGES cohort (23) (*n* = 44,642) and KNHANES (18) (*n* = 24,587), both published in 2025. These studies provide crucial evidence from non-Western populations, where dietary patterns and UPF compositions differ substantially from Western settings. The consistency of findings across Asian and Western populations strengthens the generalizability of the UPF-NAFLD association.

Fourth, we are the first to systematically discuss sex-specific differences. Two included studies (18, 23) reported stronger associations in women (female OR = 1.48–1.52; male OR = 1.06–1.35). While these findings suggest potential sex dimorphism, they are currently derived from Asian cohorts only and require confirmation in other populations. This may reflect hormonal protection against visceral adiposity in premenopausal women, which, when overwhelmed by UPF-induced metabolic stress, leads to a relatively higher risk increase. Future studies should explicitly examine effect modification by menopausal status and sex hormones.

### 4.3 . Potential mechanisms: a hierarchical view

The differential effect sizes across these exposures point to distinct mechanistic pathways.

Processed and red meat may contribute to NAFLD through multiple converging mechanisms: (1) heme iron induces oxidative stress and lipid peroxidation (33); (2) nitrites/nitrates (preservatives) are converted to N-nitroso compounds, promoting insulin resistance and hepatic inflammation (34); (3) saturated fat and cholesterol directly increase intrahepatic triglyceride accumulation (35); and (4) advanced glycation end-products (AGEs) formed during high-temperature cooking trigger inflammatory signaling (36). The strong effect size (OR = 1.70; 95% CI: 1.29–2.24) suggests additive or synergistic effects of these pathways. Recent evidence indicates that gut microbiota metabolic reprogramming may serve as a central mechanism underlying the synergistic pathogenic effects of multiple dietary components (37), providing an integrated microbiological explanation for the observed additive effects of processed meat consumption.

Sugar-sweetened beverages are thought to act primarily through fructose-driven *de novo* lipogenesis (DNL). Fructose metabolism bypasses phosphofructokinase regulation, flooding the liver with substrates for triglyceride synthesis while inhibiting fatty acid oxidation (38). A 6-month trial by Maersk et al. (39) showed that daily SSB consumption (1 L) increased liver fat by 20–30%. The strong effect size (OR = 1.50; 95% CI: 1.23–1.84) aligns with experimental evidence.

Beyond these direct pathways, emerging evidence highlights the role of the gut microbiota as a key mediator. Functional foods and dietary supplements targeting the gut microbiota have been shown to modulate metabolic dysfunction-associated fatty liver disease through multiple mechanisms (40), suggesting that microbiota-directed interventions may offer complementary strategies to mitigate UPF-related liver injury.

Total UPF intake encompasses a heterogeneous group of products, including those with strong positive associations (processed meat, SSBs) and others with less clear or potentially neutral associations. This heterogeneity may explain the relatively weaker pooled effect observed for total UPF (OR = 1.26; 95% CI: 1.16–1.37) compared with specific subgroups (OR = 1.70 for red/processed meat and 1.50 for SSBs). This underscores the need for subgroup-specific analyses rather than relying solely on total UPF estimates. The dual-pathway mechanism (direct nutrient absorption plus gut microbiota modulation) proposed for food and medicine homology components (41) offers a useful framework for understanding why different UPF types may have differential metabolic effects, although direct evidence within the UPF framework remains limited and requires further investigation.

Emerging mechanisms include gut microbiota disruption by emulsifiers and artificial sweeteners (42, 43), endocrine-disrupting chemicals from packaging (e.g., bisphenol A), and low-grade systemic inflammation as reflected by elevated C-reactive protein levels. The gut microbiota has emerged as a central node linking dietary exposures to metabolic disease, with microbiota-targeted functional foods and supplements showing promise in modulating MAFLD pathogenesis (40).

### 4.4 . Sex-specific associations: a novel finding

The finding of stronger associations in women warrants further discussion. In the KNHANES study (18), the fully adjusted OR for NAFLD in women was 1.52 (95% CI: 1.28–1.81), whereas the association in men was null (OR = 1.06; 95% CI: 0.89–1.26). Similarly, KoGES (23) reported a higher HR for women (1.48) than for men (1.35). While these findings suggest potential sex dimorphism, they are currently derived from Asian cohorts only and require confirmation in other populations. Several hypotheses may explain this sex dimorphism.

#### 4.4.1 Hormonal protection

Premenopausal women have higher insulin sensitivity and lower visceral adiposity than men due to estrogenic effects (44). However, when exposed to UPF-induced metabolic stressors (excess fructose, saturated fat, AGEs), this protective reservoir may be more rapidly depleted, leading to a proportionally greater relative risk increase.

#### 4.4.2 Fat partitioning

Women typically store fat subcutaneously, whereas men store fat viscerally. When the subcutaneous buffer capacity is exceeded (e.g., by UPF-induced caloric surplus), fat may spill over into visceral and ectopic (hepatic) depots (44). This threshold effect could make women more sensitive to dietary insults once a certain intake level is reached.

#### 4.4.3 Reporting bias

Women generally report dietary intake more accurately than men in FFQs, which may reduce non-differential misclassification and enhance the ability to detect associations. While this could contribute to the observed difference, the consistency across multiple studies suggests a genuine biological phenomenon.

#### 4.4.4 Clinical implications

These findings suggest that sex-informed dietary counseling—particularly focusing on midlife women undergoing menopausal transition—may be beneficial. Future prospective studies should collect menopausal status and sex hormone data to further elucidate these mechanisms.

### 4.5 . Heterogeneity and its sources

Moderate heterogeneity was observed across analyses (I^2^ = 53–76%). Subgroup analyses suggested that heterogeneity was partly explained by: (1) study design—cohort studies showed lower effect estimates than cross-sectional studies, likely due to better control of temporal bias; (2) NAFLD diagnostic method—studies using CAP or ultrasound showed higher effect estimates than those using FLI or HSI, reflecting the variability in diagnostic accuracy across these modalities; (3) geographic region—Asian and Western studies yielded similar results, suggesting cross-population consistency; and (4) adjustment for BMI—studies adjusting for BMI showed slightly attenuated but still significant associations, indicating that UPF effects are partially but not fully mediated by adiposity.

To further explore heterogeneity, we conducted post-hoc subgroup analyses stratified by study design. For total UPF, the pooled OR was 1.22 (95% CI: 1.10–1.36; I^2^ = 48%) in cohort studies and 1.38 (95% CI: 1.16–1.64; I^2^ = 61%) in cross-sectional studies. For red/processed meat, the corresponding estimates were 1.41 (95% CI: 1.16–1.72) in cohort studies and 1.69 (95% CI: 1.28–2.22) in cross-sectional studies. The consistently lower estimates in prospective cohort designs—which are less susceptible to recall and selection bias—suggest that the true effect size may be closer to the lower end of our confidence intervals, although the association remains significant across both designs.

### 4.6 . Mediation by adiposity

Several studies conducted mediation analyses. Liu et al. (15) reported that dietary fiber and saturated fat mediated 15–21% of the UPF-NAFLD association. Zhao et al. (17) found that BMI and waist circumference mediated 68%−71% of the association, while diabetes mediated 25%. These findings indicate that while adiposity is an important mediator, a substantial proportion of the UPF effect operates through direct metabolic pathways (e.g., fructose-induced DNL, gut microbiota dysbiosis, AGE-induced inflammation).

### 4.7 . Strengths and limitations

Strengths include: (1) first meta-analysis to systematically compare total UPF with selected food-group exposures (red/processed meat and SSBs); (2) strict inclusion criteria using only NOVA-defined UPF, excluding fast-food proxy studies; (3) inclusion of 9 recent large-scale studies (total *N* > 240,000), including 2025 Asian cohorts; (4) comprehensive analyses for red/processed meat and SSBs as selected food-group exposures; (5) rigorous quality assessment and sensitivity analyses; (6) first systematic discussion of sex-specific differences.

Limitations include: (1) observational designs preclude causal inference; (2) moderate heterogeneity (I^2^ = 53–75%), partly explained by differences in exposure assessment and NAFLD diagnostic criteria; (3) potential residual confounding despite adjustment; (4) most NAFLD diagnoses by ultrasound or non-invasive scores rather than biopsy; (5) limited number of studies for SSB food-group exposure analysis (*n* = 3); (6) lack of dose-response meta-analysis due to inconsistent reporting of exposure levels across studies (reported as % weight, % kcal, g/day, or scores with varying cutoffs), precluding meaningful harmonization; (7) sex-stratified findings from only two Asian cohorts requiring confirmation in other populations; (8) pooling of different study designs in the primary analysis, although post-hoc subgroup analyses by design were conducted; (9) Embase not searched due to institutional access limitations, partially mitigated by three major databases and manual reference screening;(10) The review protocol was not formally registered on PROSPERO, although the search strategy and analysis plan were pre-specified prior to conduct.

### 4.8 . Implications for clinical practice and public health

#### 4.8.1 Clinical practice

Our findings support a tiered dietary counseling approach: first-line should focus on reducing red/processed meat and SSBs, which show the strongest associations. Second-line should address overall UPF reduction. Simple, actionable messages (“limit sausages, bacon, ham, and sugary drinks”) may be more feasible than generic advice to “avoid ultra-processed foods,” although the comparative effectiveness of these approaches requires prospective evaluation.

#### 4.8.2 Public health policy

Existing policies targeting SSBs (e.g., sugar taxation, front-of-pack warning labels) are supported by our findings. Our results extend this to processed meats, suggesting that similar policies could be considered, although the observational nature of the underlying evidence precludes definitive causal recommendations. Given the 26% increased risk for total UPF, even moderate reductions could yield population-level benefits.

#### 4.8.3 Research priorities

Future prospective studies should: (1) use repeated dietary assessments with standardized NOVA classification; (2) employ objective NAFLD measurement (MRI or biopsy); (3) examine effect modification by sex, menopausal status, and genetic susceptibility (e.g., PNPLA3, TM6SF2 variants); (4) conduct randomized controlled trials of processed meat or SSB reduction on liver fat.

## 5 Conclusions

In this large systematic review and meta-analysis, higher total UPF intake (strictly NOVA-defined) was associated with a 26% increased NAFLD risk. Red/processed meat and sugar-sweetened beverages showed substantially stronger associations (70% and 50% increased risk, respectively).These findings suggest that dietary recommendations may be better targeted at specific high-risk food groups rather than advocating blanket avoidance of all UPFs, as the latter approach is not directly supported by the current evidence. Sex-specific differences warrant further investigation. Public health policies may consider prioritizing processed meats and SSBs, pending confirmation from higher-level evidence.

## Data Availability

The data analyzed in this study is subject to the following licenses/restrictions: The extracted data are available from the corresponding author upon reasonable request, as they are derived from previously published studies and no original data were generated. Requests to access these datasets should be directed to Chenglong Zhang, day1121@163.com.

## References

[1] Younossi ZM Koenig AB Abdelatif D Fazel Y Henry L Wymer M. Global epidemiology of nonalcoholic fatty liver disease-Meta-analytic assessment of prevalence, incidence, and outcomes. Hepatology. (2016) 64:73–84. doi: 10.1002/hep.28431 26707365

[2] Zhou F Zhou J Wang W Zhang XJ Ji YX Zhang P . Unexpected rapid increase in the burden of NAFLD in China from 2008 to 2018: a systematic review and meta-analysis. Hepatology. (2019) 70:1119–33. doi: 10.1002/hep.30702 31070259

[3] Targher G Byrne CD Lonardo A Zoppini G Barbui C. Non-alcoholic fatty liver disease and risk of incident cardiovascular disease: a meta-analysis. J Hepatol. (2016) 65:589–600. doi: 10.1016/j.jhep.2016.05.013 27212244

[4] Oddy WH Herbison CE Jacoby P Ambrosini GL O'Sullivan TA Ayonrinde OT . The Western dietary pattern is prospectively associated with nonalcoholic fatty liver disease in adolescence. Am J Gastroenterol. (2013) 108:778–85. doi: 10.1038/ajg.2013.95 23545714

[5] Riazi K Raman M Taylor L Swain MG Shaheen AA. Dietary patterns and components in Nonalcoholic Fatty Liver Disease (NAFLD): what key messages can health care providers offer? Nutrients. (2019) 11:2878. doi: 10.3390/nu11122878 31779112 PMC6950597

[6] Monteiro CA Cannon G Levy RB Moubarac JC Louzada ML Rauber F . Ultra-processed foods: what they are and how to identify them. Public Health Nutr. (2019) 22:936–41. doi: 10.1017/S1368980018003762 30744710 PMC10260459

[7] Monteiro CA Moubarac JC Cannon G Ng SW Popkin B. Ultra-processed products are becoming dominant in the global food system. Obes Rev. (2013) 14 Suppl 2:21–8. doi: 10.1111/obr.12107 24102801

[8] Zelber-Sagi S Ivancovsky-Wajcman D Fliss Isakov N Webb M Orenstein D Shibolet O . High red and processed meat consumption is associated with non-alcoholic fatty liver disease and insulin resistance. J Hepatol. (2018) 68:1239–46. doi: 10.1016/j.jhep.2018.01.015 29571924

[9] Ma J Fox CS Jacques PF Speliotes EK Hoffmann U Smith CE . Sugar-sweetened beverage, diet soda, and fatty liver disease in the Framingham Heart Study cohorts. J Hepatol. (2015) 63:462–9. doi: 10.1016/j.jhep.2015.03.032 26055949 PMC4827616

[10] Kim Y Cho Y Kim JE Lee DH Oh H. Ultra-processed food intake and risk of type 2 diabetes mellitus: a dose-response meta-analysis of prospective studies. Diabetes Metab J. (2025) 49:1064–74. doi: 10.4093/dmj.2024.0706 40490026 PMC12436045

[11] Zhang J Shu L Chen XP. Ultra-processed foods and non-alcoholic fatty liver disease: an updated systematic review and dose-response meta-analysis. Front Nutr. (2025) 12:1631975. doi: 10.3389/fnut.2025.1631975 40717999 PMC12289574

[12] Guo C Yang W Zhou J Wang JJ Ji D. Ultra-processed food intake and risk of adverse liver outcomes: a meta-analysis. J Food Sci. (2025) 90:e70303. doi: 10.1111/1750-3841.70303 40476756

[13] Page MJ McKenzie JE Bossuyt PM Boutron I Hoffmann TC Mulrow CD . The PRISMA 2020 statement: an updated guideline for reporting systematic reviews. BMJ. (2021) 372:n71. doi: 10.1136/bmj.n71 33782057 PMC8005924

[14] Higgins JPT Thomas J Chandler J Cumpston M Li T Page MJ . Cochrane *Handbook for Systematic Reviews of Interventions version 6.5* (2024). Cochrane, 2024. Available from http://www.training.cochrane.org/handbook (Accessed February 20, 2026).

[15] Liu Z Huang H Zeng Y Chen Y Xu C. Association between ultra-processed foods consumption and risk of non-alcoholic fatty liver disease: a population-based analysis of NHANES 2011-2018. Br J Nutr. (2023) 129:997–1005. doi: 10.1017/S0007114522003956 36522692

[16] Zhang YF Qiao W Zhuang J Feng H Zhang Z Zhang Y. Association of ultra-processed food intake with severe non-alcoholic fatty liver disease: a prospective study of 143073 UK Biobank participants. J Nutr Health Aging. (2024) 28:100352. doi: 10.1016/j.jnha.2024.100352 PMC12877288 39340900

[17] Zhao L Zhang X Martinez Steele E Lo CH Zhang FF Zhang X. Higher ultra-processed food intake was positively associated with odds of NAFLD in both US adolescents and adults: a national survey. Hepatol Commun. (2023) 7:e0240. doi: 10.1097/HC9.0000000000000240 37655983 PMC10476803

[18] Kim BS Park JE Kim NG. Sex differences in the association between ultra-processed food consumption and NAFLD: an analysis of KNHANES 2013-2021 data. J Clin Med. (2025) 14:7930. doi: 10.3390/jcm14227930 PMC12653714 41302966

[19] Zhang S Gan S Zhang Q Liu L Meng G Yao Z . Ultra-processed food consumption and the risk of non-alcoholic fatty liver disease in the Tianjin Chronic Low-grade Systemic Inflammation and Health Cohort Study. Int J Epidemiol. (2021) 51:237–49. doi: 10.1093/ije/dyab174 34528679

[20] Ivancovsky-Wajcman D Fliss-Isakov N Webb M Bentov I Shibolet O Kariv R . Ultra-processed food is associated with features of metabolic syndrome and non-alcoholic fatty liver disease. Liver Int. (2021) 41:2635–45. doi: 10.1111/liv.14996 34174011

[21] Emami O Nikparast A Sepehrinia M Hadi S Mirzay Razzaz J Homayounfar R. Ultra-processed food consumption and the risk of developing metabolic dysfunction-associated steatotic liver disease (MASLD): a five-year prospective cohort study in Iranian adults. J Health Popul Nutr. (2025) 44:409. doi: 10.1186/s41043-025-01150-4 41291958 PMC12648998

[22] Commins I Clayton-Chubb D Fitzpatrick JA George ES Schneider HG Phyo AZZ . Associations between MASLD, ultra-processed food and a Mediterranean dietary pattern in older adults. Nutrients. (2025) 17:1415. doi: 10.3390/nu17091415 40362724 PMC12073359

[23] Fu J Tan LJ Shin S. Consumption of ultra-processed food and risk of non-alcoholic fatty liver disease: a prospective analysis of the Korean Genome and Epidemiology Study. Mol Nutr Food Res. (2025) 69:e70099. doi: 10.1002/mnfr.70099 40346772 PMC12236129

[24] Odegaard AO Jacobs DR Jr VanWagner LB Pereira MA. Levels of abdominal adipose tissue and metabolic-associated fatty liver disease (MAFLD) in middle age according to average fast-food intake over the preceding 25 years: the CARDIA Study. Am J Clin Nutr. (2022) 116:255–62. doi: 10.1093/ajcn/nqac079 PMC9257467 35679431

[25] Zelber-Sagi S Nitzan-Kaluski D Goldsmith R Webb M Blendis L Halpern Z . Long term nutritional intake and the risk for non-alcoholic fatty liver disease (NAFLD): a population based study. J Hepatol. (2007) 47:711–7. doi: 10.1016/j.jhep.2007.06.020 17850914

[26] Peng H Xie X Pan X Zheng J Zeng Y Cai X . Association of meat consumption with NAFLD risk and liver-related biochemical indexes in older Chinese: a cross-sectional study. BMC Gastroenterol. (2021) 21:221. doi: 10.1186/s12876-021-01688-7 34001005 PMC8127290

[27] Alferink LJM Erler NS de Knegt RJ Hofman A Stricker BH Janssen HLA . Adherence to a plant-based, high-fibre dietary pattern is related to regression of non-alcoholic fatty liver disease in an elderly population. Eur J Epidemiol. (2020) 35:1069–85. doi: 10.1007/s10654-020-00627-2 PMC7695656 32323115

[28] Rahimi-Sakak F Maroof M Emamat H Hekmatdoost A. Red and processed meat intake in relation to non-alcoholic fatty liver disease risk: results from a case-control study. Clin Nutr Res. (2022) 11:42–9. doi: 10.7762/cnr.2022.11.1.42 35223680 PMC8844531

[29] Noureddin M Zelber-Sagi S Wilkens LR Porcel J Boushey CJ Le Marchand L . Diet associations with nonalcoholic fatty liver disease in an ethnically diverse population: the Multiethnic Cohort. Hepatology. (2020) 71:1940–52. doi: 10.1002/hep.30967 31553803 PMC7093243

[30] Hashemian M Merat S Poustchi H Jafari E Radmard AR Kamangar F . Red meat consumption and risk of nonalcoholic fatty liver disease in a population with low meat consumption: the Golestan Cohort Study. Am J Gastroenterol. (2021) 116:1667–75. doi: 10.14309/ajg.0000000000001229 PMC8460710 33767101

[31] Yari Z Cheraghpour M Aghamohammadi V Alipour M Ghanei N Hekmatdoost A. Energy-dense nutrient-poor snacks and risk of non-alcoholic fatty liver disease: a case-control study in Iran. BMC Res Notes. (2020) 13:214. doi: 10.1186/s13104-020-05063-9 32299509 PMC7164180

[32] Henney AE Gillespie CS Alam U Hydes TJ Cuthbertson DJ. Ultra-processed food intake is associated with non-alcoholic fatty liver disease in adults: a systematic review and meta-analysis. Nutrients. (2023) 15:2266. doi: 10.3390/nu15102266 37242149 PMC10224355

[33] Zhao Z Li S Liu G Yan F Ma X Huang Z . Body iron stores and heme-iron intake in relation to risk of type 2 diabetes: a systematic review and meta-analysis. PLoS ONE. (2012) 7:e41641. doi: 10.1371/journal.pone.0041641 22848554 PMC3406072

[34] Kim Y Keogh J Clifton P. A review of potential metabolic etiologies of the observed association between red meat consumption and development of type 2 diabetes mellitus. Metabolism. (2015) 64:768–79. doi: 10.1016/j.metabol.2015.03.008 25838035

[35] Luukkonen PK Sadevirta S Zhou Y Kayser B Ali A Ahonen L . Saturated fat is more metabolically harmful for the human liver than unsaturated fat or simple sugars. Diabetes Care. (2018) 41:1732–9. doi: 10.2337/dc18-0071 29844096 PMC7082640

[36] Hyogo H Yamagishi S Iwamoto K Arihiro K Takeuchi M Sato T . Elevated levels of serum advanced glycation end products in patients with non-alcoholic steatohepatitis. J Gastroenterol Hepatol. (2007) 22:1112–9. doi: 10.1111/j.1440-1746.2007.04943.x 17559366

[37] Wang Y Huang B Wei X Guan Y Li L. Zheng Y, et al. Gut microbiota metabolic reprogramming drives the development of metabolic diseases in the host. Gut Microbes. (2026) 18:2644681. doi: 10.1080/19490976.2026.2644681 41830551 PMC12990950

[38] Softic S Cohen DE Kahn CR. Role of dietary fructose and hepatic *de novo* lipogenesis in fatty liver disease. Dig Dis Sci. (2016) 61:1282–93. doi: 10.1007/s10620-016-4054-0 26856717 PMC4838515

[39] Maersk M Belza A Stødkilde-Jørgensen H Ringgaard S Chabanova E Thomsen H . Sucrose-sweetened beverages increase fat storage in the liver, muscle, and visceral fat depot: a 6-mo randomized intervention study. Am J Clin Nutr. (2012) 95:283–9. doi: 10.3945/ajcn.111.022533 22205311

[40] Chang Z Xu X. Xu Y, Wei X, Yang G, Guo P, et al. Potential effects of functional foods and dietary supplements on metabolic-associated fatty liver disease and the underlying mechanisms: a narrative review with a focus on the modulation of the gut microbiota. J Agric Food Chem. (2025) 73:29247–80. doi: 10.1021/acs.jafc.5c05587 41212763

[41] Zhai S Li L Jia J. Cao Y, Li M, Zheng Y, et al. Food and medicine homology components ameliorate metabolic diseases via dual pathways: blood absorption and nonabsorptive pathways. Food Med Homol. (2026) 3:9420080. doi: 10.26599/FMH.2026.9420080

[42] Chassaing B Koren O Goodrich JK Poole AC Srinivasan S Ley RE . Dietary emulsifiers impact the mouse gut microbiota promoting colitis and metabolic syndrome. Nature. (2015) 519:92–6. doi: 10.1038/nature14232 25731162 PMC4910713

[43] Suez J Korem T Zeevi D Zilberman-Schapira G Thaiss CA Maza O . Artificial sweeteners induce glucose intolerance by altering the gut microbiota. Nature. (2014) 514:181–6. doi: 10.1038/nature13793 25231862

[44] Lonardo A Nascimbeni F Ballestri S Fairweather D Win S Than TA . Sex differences in nonalcoholic fatty liver disease: state of the art and identification of research gaps. Hepatology. (2019) 70:1457–69. doi: 10.1002/hep.30626 30924946 PMC6766425

