# Supplementary material for: Total ultra-processed food intake and selected food-group exposures in relation to non-alcoholic fatty liver disease: a systematic review and meta-analysis
Source: Front Nutr. 2026 Sep 8;13:1876449. doi: 10.3389/fnut.2026.1876449 (PMC13595572; doi:10.3389/fnut.2026.1876449)
Supplement: Supplementary file 1 [file Supplementary_file_1.docx]

Supplementary Material

Effects of Creatine Supplementation on Exercise Performance, Physiological Outcomes, and Body Composition in Females Engaged in Exercise or Training: A Systematic Review and Multilevel Meta-Analysis

Supplementary Tables

Supplementary Table S1. Completed PRISMA 2020 for Abstracts checklist.

| **Section** | **Topic** | **Item** | **Checklist item** | **Location where item is reported** |
| --- | --- | --- | --- | --- |
| TITLE | Title | 1 | Identify the report as a systematic review. | Yes - title, p. 1 |
| BACKGROUND | Objectives | 2 | Provide an explicit statement of the main objective(s) or question(s) the review addresses. | Yes - Background and Objective, p. 1 |
| METHODS | Eligibility criteria | 3 | Specify the inclusion and exclusion criteria for the review. | Yes - Methods, pp. 1-2 |
| METHODS | Information sources | 4 | Specify the information sources used to identify studies and the date when each was last searched. | Yes - Methods, pp. 1-2 |
| METHODS | Risk of bias | 5 | Specify the methods used to assess risk of bias in included studies. | Yes - Methods, pp. 1-2 |
| METHODS | Synthesis of results | 6 | Specify the methods used to present and synthesize results. | Yes - Methods, pp. 1-2 |
| RESULTS | Included studies | 7 | Give the total number of included studies and participants and summarize relevant study characteristics. | Yes - Results, p. 2 |
| RESULTS | Synthesis of results | 8 | Present results for the main outcomes, including summary estimates, confidence intervals and direction of effect when meta-analysis was performed. | Yes - Results, p. 2 |
| DISCUSSION | Limitations of evidence | 9 | Provide a brief summary of limitations in the evidence, such as risk of bias, inconsistency and imprecision. | Yes - Results and Conclusion, p. 2 |
| DISCUSSION | Interpretation | 10 | Provide a general interpretation of the results and important implications. | Yes - Conclusion, p. 2 |
| OTHER | Funding | 11 | Specify the primary source of funding for the review. | No - funding information is provided in the full manuscript under “Funding” |
| OTHER | Registration | 12 | Provide the register name and registration number. | Yes - Methods, pp. 1-2; OSF DOI reported and registration identified as retrospective |

Checklist source: Page MJ, McKenzie JE, Bossuyt PM, Boutron I, Hoffmann TC, Mulrow CD, et al. The PRISMA 2020 statement: an updated guideline for reporting systematic reviews. BMJ. 2021;372:n71. Checklist adapted under CC BY 4.0.

Supplementary Table S2. Completed PRISMA 2020 checklist.

| **Section** | **Topic** | **Item** | **Checklist item** | **Location where item is reported** |
| --- | --- | --- | --- | --- |
| TITLE | Title | 1 | Identify the report as a systematic review. | Title, p. 1 |
| ABSTRACT | Abstract | 2 | See the PRISMA 2020 for Abstracts checklist. | Structured Abstract, pp. 1-2; completed abstract checklist supplied as Supplementary Table S1 |
| INTRODUCTION | Rationale | 3 | Describe the rationale for the review in the context of existing knowledge. | Introduction, pp. 2-3 |
| INTRODUCTION | Objectives | 4 | Provide an explicit statement of the objective(s) or question(s) the review addresses. | Introduction, final paragraph, p. 3 |
| METHODS | Eligibility criteria | 5 | Specify the inclusion and exclusion criteria for the review and how studies were grouped for the syntheses. | Methods: Search strategy and eligibility criteria, pp. 3-4; Statistical analysis, pp. 5-6 |
| METHODS | Information sources | 6 | Specify all databases, registers, websites, organisations, reference lists and other sources searched or consulted to identify studies. Specify the date when each source was last searched or consulted. | Methods: Search strategy and eligibility criteria, p. 3; Supplementary Table S3 |
| METHODS | Search strategy | 7 | Present the full search strategies for all databases, registers and websites, including any filters and limits used. | Supplementary Table S3 |
| METHODS | Selection process | 8 | Specify the methods used to decide whether a study met the inclusion criteria, including the number of reviewers, independent assessment and any automation tools. | Methods: Study selection and data extraction, p. 4 |
| METHODS | Data collection process | 9 | Specify the methods used to collect data from reports, including the number of reviewers, independent extraction, investigator contact and any automation tools. | Methods: Study selection and data extraction, p. 4 |
| METHODS | Data items | 10a | List and define all outcomes for which data were sought and explain how compatible results were selected. | Methods: Study selection and data extraction, pp. 4-5; Table 2, pp. 24-27 |
| METHODS | Data items | 10b | List and define all other variables for which data were sought and describe assumptions for missing or unclear information. | Methods: Study selection and data extraction, pp. 4-5; Tables 1-2, pp. 23-27 |
| METHODS | Study risk of bias assessment | 11 | Specify the methods used to assess risk of bias, including the tool(s), number of reviewers, independent assessment and any automation tools. | Methods: Risk of bias and certainty of evidence, p. 5 |
| METHODS | Effect measures | 12 | Specify the effect measure(s) used for each outcome in the synthesis or presentation of results. | Methods: Study selection and data extraction, p. 4 |
| METHODS | Synthesis methods | 13a | Describe the processes used to decide which studies were eligible for each synthesis. | Methods: Statistical analysis and Data integrity and reporting safeguards, pp. 5-6; Table 2 |
| METHODS | Synthesis methods | 13b | Describe methods used to prepare data for presentation or synthesis, including handling of multi-arm studies and multiple outcomes. | Methods: Study selection and data extraction and Statistical analysis, pp. 4-6 |
| METHODS | Synthesis methods | 13c | Describe methods used to tabulate or visually display results of individual studies and syntheses. | Methods: Statistical analysis, pp. 5-6; Tables 2-4; Figures 3-5 |
| METHODS | Synthesis methods | 13d | Describe methods used to synthesize results and the rationale for each choice. For meta-analysis, describe the model, heterogeneity methods and software. | Methods: Statistical analysis, pp. 5-6 |
| METHODS | Synthesis methods | 13e | Describe methods used to explore possible causes of heterogeneity. | Methods: Statistical analysis, p. 6 |
| METHODS | Synthesis methods | 13f | Describe sensitivity analyses used to assess robustness. | Methods: Statistical analysis, p. 6 |
| METHODS | Reporting bias assessment | 14 | Describe methods used to assess risk of bias due to missing results in a synthesis. | Methods: Statistical analysis, p. 6 |
| METHODS | Certainty assessment | 15 | Describe methods used to assess certainty or confidence in the body of evidence. | Methods: Risk of bias and certainty of evidence, p. 5 |
| RESULTS | Study selection | 16a | Describe the results of the search and selection process, ideally with a flow diagram. | Results: Study selection, pp. 6-7; Figure 1 |
| RESULTS | Study selection | 16b | Cite studies that might appear to meet the inclusion criteria but were excluded, and explain why they were excluded. | Figure 1 gives full-text exclusion reasons; detailed screening decisions are available in the OSF |
| RESULTS | Study characteristics | 17 | Cite each included study and present its characteristics. | Results: Characteristics of the systematic-review evidence base, pp. 7-8; Table 1, pp. 23-24 |
| RESULTS | Risk of bias in studies | 18 | Present assessments of risk of bias for each included study. | Results: Risk of bias, p. 8; Figure 2 |
| RESULTS | Results of individual studies | 19 | For all outcomes, present study-level summary results and effect estimates with precision where appropriate. | Results: Narrative synthesis, pp. 7-8; Table 2, pp. 24-27; Figures 3-4 |
| RESULTS | Results of syntheses | 20a | For each synthesis, summarize characteristics and risk of bias among contributing studies. | Results: Risk of bias and Studies contributing to the primary meta-analysis, pp. 8-9; Tables 2-3 |
| RESULTS | Results of syntheses | 20b | Present all statistical syntheses, including summary estimates, precision, heterogeneity and direction of effect. | Results: Primary multilevel meta-analysis and Domain-specific syntheses, pp. 9-10; Tables 3-4; Figures 3-4 |
| RESULTS | Results of syntheses | 20c | Present investigations of possible causes of heterogeneity. | Results: Domain-specific syntheses, p. 9; Table 4 |
| RESULTS | Results of syntheses | 20d | Present all sensitivity analyses used to assess robustness. | Results: Sensitivity analyses and certainty of evidence, p. 10; Table 4 |
| RESULTS | Reporting biases | 21 | Present assessments of risk of bias due to missing results for each synthesis assessed. | Results: Sensitivity analyses and certainty of evidence, p. 10; Figure 5 |
| RESULTS | Certainty of evidence | 22 | Present certainty or confidence assessments for each outcome assessed. | Results: Sensitivity analyses and certainty of evidence, p. 10; Table 5 |
| DISCUSSION | Discussion | 23a | Provide a general interpretation of the results in the context of other evidence. | Discussion, pp. 10-13 |
| DISCUSSION | Discussion | 23b | Discuss limitations of the evidence included in the review. | Discussion: Certainty and methodological limitations, pp. 12-13 |
| DISCUSSION | Discussion | 23c | Discuss limitations of the review processes used. | Discussion: Certainty and methodological limitations, pp. 12-13 |
| DISCUSSION | Discussion | 23d | Discuss implications for practice, policy and future research. | Discussion: Implications for future research, pp. 13-14; Conclusion, pp. 13-14 |
| OTHER INFORMATION | Registration and protocol | 24a | Provide registration information, including register name and registration number, or state that the review was not registered. | Methods: Design and reporting, p. 3 |
| OTHER INFORMATION | Registration and protocol | 24b | Indicate where the review protocol can be accessed, or state that a protocol was not prepared. | Methods: Design and reporting, p. 3; OSF DOI reported |
| OTHER INFORMATION | Registration and protocol | 24c | Describe and explain amendments to information provided at registration or in the protocol. | Methods: Design and reporting, p. 3; registration is identified as retrospective, so no prospective protocol amendments are claimed |
| OTHER INFORMATION | Support | 25 | Describe sources of financial or non-financial support and the role of funders or sponsors. | Funding, p. 14 |
| OTHER INFORMATION | Competing interests | 26 | Declare competing interests of the review authors. | Conflict of Interest, p. 14 |
| OTHER INFORMATION | Availability of data, code and other materials | 27 | Report which review materials are publicly available and where they can be found. | Data Availability Statement, p. 14; OSF DOI reported |

Checklist source: Page MJ, McKenzie JE, Bossuyt PM, Boutron I, Hoffmann TC, Mulrow CD, et al. The PRISMA 2020 statement: an updated guideline for reporting systematic reviews. BMJ. 2021;372:n71. Checklist adapted under CC BY 4.0.

**Note:** Reporting locations refer to the submitted, line-numbered manuscript accompanying this checklist, before journal typesetting. Page and line numbers may change during production; section headings and table or figure numbers provide stable locations.

Supplementary Table S3. Database-specific implementation of the search strategy.

| Database | Search date | Field/interface used | Database-specific search string | Limits | Records retrieved |
| --- | --- | --- | --- | --- | --- |
| PubMed | 5 March 2026 | PubMed Advanced Search; automatic term mapping across PubMed fields | ((creatine) AND (woman OR women OR female)) AND (exercise OR performance OR endurance OR aerobic OR strength OR power OR anaerobic OR physique OR "body composition") | 1 Jan 1996 to 5 Mar 2026; English language; journal article | 3090 |
| Scopus | 5 March 2026 | Article title, abstract and keywords | TITLE-ABS-KEY(creatine) AND TITLE-ABS-KEY(woman OR women OR female) AND TITLE-ABS-KEY(exercise OR performance OR endurance OR aerobic OR strength OR power OR anaerobic OR physique OR "body composition") | 1 Jan 1996 to 5 Mar 2026; English language; article | 8006 |
| Web of Science | 5 March 2026 | Web of Science Core Collection; Topic | TS=(creatine) AND TS=(woman OR women OR female) AND TS=(exercise OR performance OR endurance OR aerobic OR strength OR power OR anaerobic OR physique OR "body composition") | 1 Jan 1996 to 5 Mar 2026; SCI-EXPANDED, SSCI and ESCI; English language; article | 1384 |
| SPORTDiscus | 5 March 2026 | EBSCOhost; TX All Text | TX creatine AND TX (woman OR women OR female) AND TX (exercise OR performance OR endurance OR aerobic OR strength OR power OR anaerobic OR physique OR "body composition") | 1 Jan 1996 to 5 Mar 2026; English language; peer-reviewed article | 401 |
| CINAHL | 5 March 2026 | EBSCOhost; TX All Text | TX creatine AND TX (woman OR women OR female) AND TX (exercise OR performance OR endurance OR aerobic OR strength OR power OR anaerobic OR physique OR "body composition") | 1 Jan 1996 to 5 Mar 2026; English language; peer-reviewed article | 363 |
| Total | - | - | - | - | 13244 |

**Note:** Searches were executed on 5 March 2026 and limited to records published from 1 January 1996 to 5 March 2026. The same core concept was translated into each database interface. No extra team-sport or high-intensity-interval terms were added. Record counts are pre-deduplication. The PubMed row reproduces the query entered in the search interface.
