# Supplementary material for: Total ultra-processed food intake and selected food-group exposures in relation to non-alcoholic fatty liver disease: a systematic review and meta-analysis
Source: Front Nutr. 2026 Sep 8;13:1876449. doi: 10.3389/fnut.2026.1876449 (PMC13595572; doi:10.3389/fnut.2026.1876449)
Supplement: Supplementary file 2 [file Supplementary_file_2.docx]

Supplementary Table S1. Detailed characteristics of all included studies (24 studies)

| First Author, Year | Country | Study Design | Sample Size (n) | NAFLD Cases (n) | UPF Assessment Method | NAFLD Diagnostic Criteria | Effect Estimate (95% CI) | Adjustment Covariates | Quality Score |
| --- | --- | --- | --- | --- | --- | --- | --- | --- | --- |
| Total UPF | | | | | | | | | |
| Zhang S, 2021 | China | Prospective cohort | 16,168 | 3,752 | FFQ, NOVA (% g) | Ultrasound | HR 1.18 (1.07–1.30) | Age, sex, BMI, smoking, alcohol, education, occupation, income, PA, energy, family history, depression, diet quality, hypertension, dyslipidemia, diabetes | NOS 9 |
| Ivancovsky-Wajcman D, 2021 | Israel | Cross-sectional | 789 | 305 | FFQ, NOVA (% kcal) | Ultrasound | OR 1.12 (0.78–1.59) | Age, sex, BMI, SFA, protein, PA, coffee, fiber | AHRQ 7 |
| Liu Z, 2023 | USA | Cross-sectional | 6,545 | 2,224 | 24h recall, NOVA (% weight) | FLI ≥30 | OR 1.83 (1.33–2.53) | Age, sex, race, education, income, marital status, smoking, BMI, ALT, TG, total cholesterol, uric acid | AHRQ 8 |
| Zhang YF, 2024 | UK | Prospective cohort | 143,073 | 1,445 | 24h recall, NOVA (g/day) | Hospital records (ICD-10) | HR 1.26 (1.11–1.43) | Age, sex, race, education, income, smoking, alcohol, PA, BMI | NOS 8 |
| Emami O, 2025 | Iran | Prospective cohort | 5,058 | 562 | FFQ, NOVA (g/day) | CAP ≥238 dB/m + metabolic criteria | OR 2.02 (1.23–3.32) | Age, sex, FMI, PA, wealth index, HOMA-IR, TG, total energy, fruit/veg, SFA, trans fat | NOS 8 |
| Zhao L, 2023 | USA | Cross-sectional | 2,734 | 1,053 | 24h recall, NOVA (g/day, energy-adjusted) | CAP ≥285 dB/m | OR 1.72 (1.01–2.93) | Age, sex, race, education, smoking, alcohol, PA, total energy | AHRQ 8 |
| Commins I, 2025 | Australia | Cross-sectional | 6,753 | 1,963 | FFQ, NOVA (score, tertiles) | FLI ≥60 | RR 1.13 (1.05–1.22) | Age, sex, diabetes, hypertension, CKD, frailty index, education | AHRQ 8 |
| Kim BS (KNHANES), 2025 | Korea | Cross-sectional | 24,587 | ~5,124 | 24h recall, NOVA (% weight) | HSI (sex-specific) | OR 1.24 (1.10–1.41) | Age, sex, education, income, smoking, alcohol, PA, total energy | AHRQ 8 |
| KoGES, 2025* | Korea | Prospective cohort | 44,642 | 1,562 | FFQ, NOVA (g/day) | FLI ≥60 | HR 1.35 (1.06–1.71)* | Age, total energy, processed/unprocessed food intake, income, education, PA, alcohol, smoking, sleep, stress, obesity, diabetes, hypertension, dyslipidemia | NOS 8 |
| Red/processed meat | | | | | | | | | |
| Zelber-Sagi S, 2007 | Israel | Cross-sectional | 349 | 108 | FFQ (meat protein) | Ultrasound | OR 3.60 (1.70–7.80) | Age, sex, BMI, total energy | AHRQ 8 |
| Peng H, 2021 | China | Cross-sectional | 1,594 | 523 | FFQ (red meat) | Ultrasound | OR 1.72 (1.21–2.42) | Age, sex, BMI, smoking, tea intake, PA, hypertension, dyslipidemia, diabetes | AHRQ 8 |
| Alferink LJM, 2019 | Netherlands | Cross-sectional | 3,882 | 1,337 | FFQ (animal protein) | Ultrasound | OR 1.54 (1.20–1.98) | Age, sex, education, smoking, alcohol, PA, DHDI, diabetes, metabolic syndrome, cholesterol | AHRQ 8 |
| Rahimi-Sakak F, 2022 | Iran | Case-control | 999 | 196 | FFQ (processed meat) | Ultrasound + Fibroscan | OR 3.25 (1.57–6.73) | Age, sex, BMI, total energy, diet quality, diabetes, smoking, PA | NOS 8 |
| Noureddin M, 2020 | USA | Nested case-control | 32,448 | 2,974 | FFQ (processed meat) | Medicare claims | OR 1.18 (1.05–1.32) | BMI, alcohol, coffee, PA, energy | NOS 9 |
| Hashemian M, 2021 | Iran | Prospective cohort | 50,045 | 3,218 | FFQ (red meat) | Ultrasound | OR 1.59 (1.06–2.38) | Age, sex, BMI, smoking, PA, energy, SES | NOS 8 |
| Sugar-sweetened beverages | | | | | | | | | |
| Ma J, 2015 | USA | Cross-sectional | 2,634 | ~448 | FFQ (SSB ≥1/d) | CT liver attenuation | OR 1.61 (1.04–2.49) | Age, sex, smoking, cohort, energy, alcohol, fiber, fat, protein, diet soda, BMI | AHRQ 7 |
| Yari Z, 2020 | Iran | Case-control | 614 | 143 | FFQ (soft drinks) | Ultrasound + Fibroscan | OR 1.64 (0.82–2.65) | Age, sex, BMI, PA, alcohol, energy | NOS 6 |
| Zelber-Sagi S, 2007 | Israel | Cross-sectional | 349 | 108 | FFQ (soft drinks) | Ultrasound | OR 1.45 (1.13–1.85) | Age, gender, BMI, total calories | AHRQ 8 |

*KoGES used male HR as conservative estimate for total UPF; the paper reported sex-specific HRs (male 1.35, female 1.48).

Abbreviations: PA, physical activity; SFA, saturated fatty acids; FMI, fat mass index; FLI, Fatty Liver Index; HSI, Hepatic Steatosis Index; CAP, Controlled Attenuation Parameter; DHDI, Dutch Healthy Diet Index; SES, socioeconomic status.

Supplementary Table S2. Newcastle-Ottawa Scale (NOS) and AHRQ Quality Assessment Scores

| Study | Selection (max 4) | Comparability (max 2) | Outcome/Exposure (max 3) | Total Score (max 9) | Quality |
| --- | --- | --- | --- | --- | --- |
| Cohort/case-control (NOS) | | | | | |
| Zhang S, 2021 | 4 | 2 | 3 | 9 | High |
| Zhang YF, 2024 | 4 | 2 | 2 | 8 | High |
| Emami O, 2025 | 3 | 2 | 3 | 8 | High |
| KoGES, 2025 | 4 | 2 | 2 | 8 | High |
| Rahimi-Sakak F, 2022 | 3 | 2 | 3 | 8 | High |
| Noureddin M, 2020 | 4 | 2 | 3 | 9 | High |
| Hashemian M, 2021 | 4 | 2 | 2 | 8 | High |
| Yari Z, 2020 | 3 | 1 | 2 | 6 | Moderate |
| Cross-sectional (AHRQ, max 11) | AHRQ Score | | | |  |
| Ivancovsky-Wajcman D, 2021 | 7 | | | | High |
| Liu Z, 2023 | 8 | | | | High |
| Zhao L, 2023 | 8 | | | | High |
| Commins I, 2025 | 8 | | | | High |
| Kim BS (KNHANES), 2025 | 8 | | | | High |
| Zelber-Sagi S, 2007 | 8 | | | | High |
| Peng H, 2021 | 8 | | | | High |
| Alferink LJM, 2019 | 8 | | | | High |
| Ma J, 2015 | 7 | | | | High |

NOS scores range 0–9; high quality defined as ≥7. AHRQ scores range 0–11; high quality defined as ≥7.

Supplementary Table S3. Leave-one-out Sensitivity Analysis for Red/Processed Meat and Sugar-Sweetened Beverages

| A. Red/Processed Meat (6 studies) | | | |
| --- | --- | --- | --- |
| Excluded study | Pooled OR (95% CI) | I² (%) | P-value |
| None | 1.70 (1.29–2.24) | 76 | <0.001 |
| Zelber-Sagi 2007 | 1.56 (1.24–1.95) | 65 | <0.001 |
| Peng 2021 | 1.68 (1.26–2.25) | 77 | <0.001 |
| Alferink 2019 | 1.72 (1.26–2.35) | 78 | <0.001 |
| Rahimi-Sakak 2022 | 1.62 (1.27–2.08) | 69 | <0.001 |
| Noureddin 2020 | 1.78 (1.33–2.38) | 73 | <0.001 |
| Hashemian 2021 | 1.70 (1.26–2.30) | 78 | <0.001 |
| B. Sugar-Sweetened Beverages (3 studies) | | | |
| Excluded study | Pooled OR (95% CI) | I² (%) | P-value |
| None | 1.50 (1.23–1.84) | 0 | < 0.001 |
| Ma 2015 | 1.48 (1.18–1.86) | 0 | 0.001 |
| Yari 2020 | 1.49 (1.21–1.84) | 0 | < 0.001 |
| Zelber-Sagi 2007 | 1.63 (1.06–2.50) | 0 | 0.026 |
